# Extreme Weather Events, Air Pollution, and Cardiovascular Health: Mechanisms and Challenges

**DOI:** 10.31083/RCM47388

**Published:** 2026-04-21

**Authors:** Shiying Xu, Rong Li, Jingying Li, Zaiyong Zheng, Yanqiu Luo, Jiali Gao

**Affiliations:** ^1^Department of Pharmacy, Luzhou People's Hospital, 646000 Luzhou, Sichuan, China; ^2^Department of Cardiology, Key Laboratory of Medical Electrophysiology, Ministry of Education; Basic Medicine Research Innovation Center for cardiometabolic diseases, Ministry of Education; The Affiliated Hospital of Southwest Medical University, Southwest Medical University, 646000 Luzhou, Sichuan, China

**Keywords:** extreme weather, cardiovascular diseases, environment and public health

## Abstract

Extreme weather events and air pollution are becoming increasingly frequent and severe, posing complex challenges to human health. Although the adverse effects of these environmental stressors on cardiovascular disease are widely acknowledged, the underlying mechanisms remain incompletely understood. This review examines these mechanisms from a physiological perspective, considering both pathways that act directly on the heart and those mediated through non-cardiac organs. Airborne particulate pollutants from dust storms, wildfires, and haze days can pass through the respiratory system and reach the heart, resulting in cardiac injury. Meanwhile, extreme weather events, including heatwaves, cold spells, hurricanes, and even earthquakes, can increase cardiovascular disease risk by affecting traditional risk factors, autonomic nervous system responses, oxidative stress, inflammation, coagulation disturbances, and behavioral factors. Hence, by providing a comprehensive overview, this review aims to enhance our understanding of the cardiovascular risks associated with climate hazards.

## 1. Introduction

Extreme weather events, including heatwaves, cold spells, air pollution, dust storms, hurricanes, and wildfires, are becoming increasingly frequent and intense, posing a significant threat to human health. Current evidence suggests that the number of disasters linked to extreme weather events has increased fivefold [1]. The World Meteorological Organization (WMO) reports that extreme temperatures above 40 °C and even 50 °C are becoming more common globally [2]. Early studies have identified a correlation between elevated temperatures, carbon monoxide air pollution, and increased hospital admissions for myocardial infarction [3]. Recent longitudinal studies have revealed extreme weather events as a chronic cardiovascular disease (CVD) risk multiplier. Besides, the cardiovascular impact of climate-related events, such as hurricanes, can last for extended periods. Six years after Hurricane Katrina, admissions for acute myocardial infarction (AMI) reportedly remain elevated [4]. However, despite growing epidemiological evidence, the precise mechanisms by which extreme weather events impact cardiovascular health remain incompletely understood. This review aimed to synthesize current knowledge by examining both epidemiological trends and mechanistic insights. These mechanisms are categorized into those involving pathways that directly reach the heart and those affecting non-cardiac organs, a classification based on whether environmental stressors directly infiltrate cardiac tissues. Furthermore, case studies of successful interventions, such as air quality improvements, that have mitigated CVD risk by reducing the frequency or severity of extreme weather events, are presented. Finally, the current research challenges and future directions in this rapidly evolving field are discussed.

## 2. Mechanisms Linking Extreme Weather Events and Air Pollution to CVD

It is widely reported that cardiovascular health is affected by extreme weather and air pollution [5, 6]. For instance, a study conducted in the USA found that each additional heatwave day was associated with a 0.12% increase in monthly cardiovascular mortality [7]. Global meta-analyses have indicated a 3.44% increase in CVD_S_ mortality per 1 °C temperature rise [8], while emerging evidence has linked long-term PM2.5 exposure to accelerated atherosclerosis [9]. However, the mechanism is still unclear. Unlike external organs such as the skin, lungs, or gastrointestinal tract, the cardiovascular system comprises internal organs that are not directly exposed to the environment, making the mechanisms more complex and intertwined with each other. To discuss these mechanisms clearly, we classify these mechanisms into direct pathways to the heart and indirect pathways via non-cardiac organs (Table 1). Direct pathways involve harmful substances (e.g., PM2.5, microbes) that enter the body and directly affect the heart and vasculature. In contrast, the indirect pathways include systemic or behavioral mediators, such as temperature-induced hypertension, autonomic nervous dysfunction, or changes in lifestyle behaviors triggered by disasters.

**Table 1.** **Elaboration of pathways of extreme weather events on cardiovascular health**.

Type	Definition	Key factors/media	Main mechanisms	Cardiovascular impacts (for example)
Directly reaching the heart	Physical substances directly enter the cardiovascular system, causing cellular or tissue damage.	Particulate matter, pathogenic microorganisms, heavy metals.	Endothelial cell damage, thrombus formation, atherosclerosis, ion channel dysfunction, arrhythmias, local inflammatory response, local oxidative stress, mitochondrial dysfunction.	Myocardial infarction, ischemic heart disease, arrhythmias, myocardial damage, rheumatic fever, cardiac implantable electronic device infection.
Pathways affecting non-cardiac organs	Influences something else first before acting on a cardiovascular mechanism.	Heatwave, cold waves, hurricanes, wildfires, dust storms, air pollution.	Worsening of traditional risk factors (diabetes, obesity, hypertension, dyslipidemia), autonomic nervous system dysfunction, systemic oxidative stress, systemic inflammatory responses, coagulation and anticoagulation system disorders, altered human behavior (smoking, alcohol consumption, drug abuse, poor adherence, reduced exercise), abnormal cardiac development.	Increased AMI admissions, hypertension, stress-induced cardiomyopathy, arrhythmias, atherosclerosis, thrombosis, and congenital heart disease.

AMI, acute myocardial infarction.

## 3. Cardiac Exposure via Blood Circulation: Environmental Particulates and Pathogens

Physical environment contaminants can reach the heart through inhalation and subsequent entry into the blood circulation.

### 3.1 Direct Cardiac Exposure to Environmental Particulates

During dust storms, wildfires, and ambient air pollution, a significant quantity of fine particles is generated and suspended in the air. These particles can enter the lungs and reach the heart via the bloodstream [10, 11]. A postmortem report indicated that the concentration of iron-based particles in the left ventricle was significantly higher in young adults who lived in high-pollution areas than in those who lived in low-pollution areas [12]. Particles can cross the endothelial cells and reach the intercellular clefts of cardiac myocytes. Subsequently, these particles attach to the cell membranes of cardiac myocytes and are internalized through endocytosis [13, 14]. During each of these processes, particles can have detrimental effects on cardiac myocytes. First, particles can injure vascular endothelial cells, leading to thrombosis and atherosclerosis, which in turn promote the occurrence of myocardial infarction and ischemic heart disease. These particles can also attach to the membrane of cardiomyocytes. Unlike other cells, ion channels on the cell membrane play a crucial role in maintaining the normal electrical activity of the heart. However, literature reports have indicated that particles at the nanoscale can disrupt the ion channels and lead to arrhythmia [14, 15, 16]. This mechanism may partly explain the increase in cardiac arrhythmia following exposure to air pollution [17, 18, 19]. Furthermore, as a foreign substance, these particles can induce an inflammatory response in cardiac tissues, which may finally lead to cardiac injury. Particles emitted by diesel trucks have been proven to induce vascular inflammatory response *in vitro* [20]. Other artificial particles, especially metal-based nanoparticles, have also been reported to induce inflammation in heart tissues [21, 22]. Following internalization, particles can generate excessive reactive oxygen species (ROS) due to their high surface area and chemical reactivity, leading to oxidative stress, an effect demonstrated in both cellular and animal models [23, 24]. In addition, ambient particles can disturb the normal function of cardiomyocyte organelles such as mitochondria, which may result in cell death [12, 25]. For example, PM2.5 can increase mitochondrial ROS, which ultimately leads to apoptosis and heart defects in zebrafish [26].

### 3.2 Direct Cardiac Exposure to Pathogens

Pathogenic microorganisms, including bacteria, represent another class of ambient substances capable of reaching the heart. Extreme weather events may increase the concentration of pathogenic microorganisms in the ambient air. These microorganisms can be suspended in the air with particles and enter the human body through breathing. In Ethiopian children, acute rheumatic fever demonstrates seasonal fluctuations, with group A beta-hemolytic streptococci bacteria predominating during hot and humid seasons [27]. Research in France has revealed that the risk of infections of cardiac implantable electronic devices is higher with high temperatures and precipitation [28].

In summary, physical agents associated with extreme weather events can enter human body and reach cardiac tissue, where they may induce endothelial cell damage, ion channel dysfunction, arrhythmias, local inflammatory response, and oxidative stress. These mechanisms have been thoroughly reviewed in several prior studies [29, 30, 31, 32, 33].

## 4. Pathways Affecting Non-Cardiac Organs

As an internal organ, the heart is generally protected from direct exposure to most external environmental factors, except for particulate matter and certain inhaled agents. However, as the central organ of the circulatory system, it is intricately regulated by multiple physiological systems, such as the autonomic nervous system, endocrine signaling, and thermoregulatory mechanisms, that enable the body to adapt to varying environmental conditions. Extreme weather events can disrupt these regulatory systems, leading to systemic imbalances that indirectly impair cardiovascular function and contribute to disease development.

### 4.1 Amplification of Traditional Cardiovascular Risk Factors by Extreme Weather Events and Air Pollution

Traditional risk factors, such as diabetes, obesity, hypertension, and hyperlipidemia, significantly increase the incidence of cardiovascular disease. The pathological mechanism of these diseases usually affects the whole body, including the heart. Among patients with diabetes, high blood glucose alters the cardiac energy metabolic pattern [34], increases fatty acid oxidation, upregulates the production of acetyl coenzyme A and citrate, reduces ATP production and causes the excessive generation of ROS. Furthermore, diabetes disrupts excitation-contraction coupling by impairing Ca^2+^ reuptake and release, ultimately resulting in diastolic and systolic dysfunction [35]. On the one hand, extreme weather events such as heatwaves, cold spells, hurricanes [36], as well as air pollution [37, 38], can affect the occurrence and development of diabetes [39, 40]. On the flip side, individuals with diabetes are particularly vulnerable to the impacts of extreme weather events and are at a heightened risk of developing CVD when exposed to extreme climate conditions. For example, in Taiwan, the mortality rate of individuals with previous ischemic heart disease is higher among people with diabetes than among those without diabetes after exposure to hurricanes [41]. A time series study conducted in Hong Kong revealed that after exposure to extreme temperatures, diabetes patients showed a higher risk of AMI admissions than patients without diabetes [42]. Collectively, these results indicate that individuals with diabetes and CVDs are more vulnerable to the health effects of extreme weather events.

Similar to diabetes, hyperlipidemia also alters cardiac energy metabolism [43]. In addition to this effect, elevated blood cholesterol can directly deposit on the vessel wall, leading to the development of arterial atherosclerosis, especially after vascular endothelial injury (by particulate matter, nicotine, etc.). A multicenter longitudinal cohort study explored the association between hyperlipidemia starting in young adulthood and the risk of CVD. The results indicated that cumulative exposure to low-density lipoprotein cholesterol and triglycerides was independently associated with CVD risk [44], may exacerbate the association of air pollutants and CVD. A large, population-based investigation in China revealed that hyperbetalipoproteinemia showed the strongest association with air pollution among cardiometabolic factors. Participants with hyperbetalipoproteinemia and high ambient PM1.0 levels showed a higher prevalence of CVDs (odds ratio (OR) 3.19, 95% confidence interval (CI), 2.43–4.18) [45].

Hypertension is another common risk factor for CVD, characterized by high blood pressure, which leads to pressure overload on the left ventricle. To adapt to increased ventricular pressure and maintain high cardiac output, mediators such as hormones, growth factors, and cytokines are secreted to remodel the ventricular wall [46]. Unfortunately, these mediators can also be harmful to the heart, leading to cardiac hypertrophy and heart failure. Hypertension is determined by cardiac output and vascular wall tension, which are also the primary effectors of the stress response when an individual experiences extreme changes. After Hurricane Katrina, the blood pressure in the affected area was significantly increased [47]. Acute exposure to air pollution [48], noise [49], cold spells [50], and other factors have been found to elevate blood pressure. Furthermore, long-term exposure to these extreme weather events has been linked to the development of hypertension and CVD.

### 4.2 Neuroautonomic Dysregulation Induced by Extreme Weather Events and Air Pollution

Environmental changes can affect cardiac health through the autonomic nervous system. As the brain cannot regulate the heart through direct conscious or motor commands, it exerts control indirectly through the autonomic nervous system. Furthermore, the autonomic nervous system regulates physical functions in response to external stimuli. As the main effector organ of the autonomic nervous system, heart rate variability has been used to measure the activity of the autonomic nervous system, which contains parasympathetic and sympathetic fibers. Under normal conditions, sympathetic innervation increases heart activity while parasympathetic innervation inhibits heart activity. The dynamic balance between parasympathetic and sympathetic activity maintains the physiological activity of the heart.

Transient activation of the sympathetic nervous system can increase the output of heart, which is beneficial for escaping immediate threats. However, consistent activation of the sympathetic system is harmful, resulting in oxidative stress, cell apoptosis, ventricular remodeling, and arrhythmia. Many extreme environmental changes can lead to autonomic nervous system dysfunction. Acute environmental changes, such as earthquakes, dust storms, hurricanes, and wildfires, are typically related to life-threatening conditions. This stress response activates the sympathetic nervous system to avoid injury. Acute emotional changes, such as fear, anger, and euphoria, can also induce a sympathetic response [51]. After an earthquake, residents were observed to have increased blood pressure that remained consistently high for one week [52, 53], with some experiencing reversible left ventricular dysfunction known as Takotsubo syndrome [54]. It is widely accepted that the sympathetic nervous system contributes to these cardiovascular changes to some extent [55].

After exposure to cold temperatures, the sympathetic nervous system is activated, accompanied by an increase in blood pressure [56, 57]. Among volunteers, cold air exposure increased angiotensin II and norepinephrine levels and stimulated the renin-angiotensin system. Furthermore, the cardiac injury markers cardiac troponin I and muscle myoglobin were elevated after exposure to cold air. This indicates that cold air exposure not only increases the activation of the sympathetic nervous system but also causes cardiac injury [58]. Similarly, heatwave exposure disrupts the autonomic nervous system. The heart rate and cardiac output are increased to dissipate heat after exposure to heatwaves [59]. Researchers found that approximately 40% of the increase in heart rate was due to autonomic nervous system activity, with 25% caused by sympathetic activation and 75% induced by parasympathetic withdrawal [60]. Unfortunately, the ability of older adults to adapt to high ambient temperatures has decreased [61]. This may partly explain why older adults are more vulnerable after exposure to heatwaves. However, the extent to which the autonomic nervous system contributes to this vulnerability remains unknown [62].

Besides, acute emotional stress can activate the sympathetic nervous system. Among die-hard sports fans, sports match events can stimulate the sympathetic nervous system and result in adverse cardiovascular effects, especially for the defeated team [63, 64]. Furthermore, short-term exposure to fine particles and ozone can disrupt the clearance of norepinephrine, resulting in effects similar to excessive activation of the sympathetic nervous system [65, 66]. Besides acute activation, chronic activation of the sympathetic nervous system is also deleterious to cardiovascular health. Both animal and human experiments have shown that the concentration of norepinephrine and dopamine significantly increases after long-term exposure to air pollution particles [67, 68, 69]. Fear [51], anxiety, and intense emotional changes can induce sympathetic responses through the brain’s cortical regions. Individuals with posttraumatic stress disorder (PTSD) may re-experience the fear they felt during the traumatic event. The re-experiencing of fearful memories can lead to chronic and persistent activation of the sympathetic nervous system [70, 71, 72]. Current reports suggest that sympatholytic medications, such as propranolol, can alleviate the symptoms of PTSD [73, 74]. This may explain the higher risk of PTSD and cardiovascular disease even a decade after Hurricane Katrina in populations from affected areas, especially at night and on weekends [75]. After the earthquake, a higher risk of CVDs was observed alongside PTSD among disaster victims [76, 77, 78]. Unfortunately, recent research has reported that a high heart rate, in turn, can induce anxiety [79]. This indicates that individuals with fast heart rhythm disorder and anxiety may become caught in a mutually reinforcing cycle of misconduct, where the sympathetic nervous system plays a critical role.

Inhaled air pollutants directly activate the sensory nervous system in the respiratory system, which subsequently activates pulmonary sensor nerve fibers, including C-nerve fibers, rapidly adapting pulmonary receptors, and slowly adapting pulmonary receptors. On the one hand, stimulus signals lead to coughing, bronchoconstriction, and dyspnea to remove air pollutants. On the other hand, the stimulation disrupts the autonomic nervous system. The activation of slowly adapting pulmonary receptors can also lead to ventricular arrhythmias. Furthermore, baroreceptors and chemoreceptors can be activated by air pollutants, which are critical receptors of the autonomic nervous system that maintain normal blood pressure.

### 4.3 Systemic Oxidative Stress Triggered by Extreme Weather Events and Air Pollution

Oxidative stress plays an important role in the development of cardiovascular diseases such as atherosclerosis, ischemic heart disease, myocardial infarction, and heart failure. Oxidative stress occurs when the generation of ROS exceeds the antioxidant capacities. Excessive ROS attacks normal cellular components, leading to protein oxidation, DNA oxidation, and lipid peroxidation.

Environmental change induces systemic oxidative stress through three main mechanisms. First, air pollutants such as fine particles, ozone, nitrogen oxides, and reactive organic compounds have high oxidative and pro-oxidative capacities, which promote the generation of ROS. Besides, inhalation of SO_2_ can inhibit the activity of cytochrome c oxidase (COX), which is essential for the mitochondrial electron transport chain, resulting in excessive ROS generation [80]. An *in vivo* study confirmed that the marker of oxidative stress increased after exposure to higher indoor air pollution [81]. Interestingly, researchers have reported that exposure to PM2.5 can lead to changes in microRNAs present in extracellular vesicles among human volunteers. Intriguingly, some of these altered microRNAs have been associated with oxidative stress [82].

The transition metal can also induce excess ROS through Fenton and Fenton-like chemical reactions. In human cardiomyocytes, iron particles can release Fe ions (Fe^2+^), which react with H_2_O_2_ to generate hydroxyl radicals (⋅OH), a main type of ROS, and Fe^3+^. Besides, other transition metals found in polluted air, such as copper (Cu) [83], nickel (Ni), and zinc (Zn), can trigger excessive ROS production through the Fenton reaction. A field trial revealed that ROS levels significantly increased in participants exposed to humid room conditions, and dehumidification reversed this trend [84]. In hypertensive rats, the level of oxidative stress significantly increased after exposure to ozone compared to the control group. Furthermore, oxidative stress is exacerbated after co-exposure to heatwave weather and ozone [85].

### 4.4 Systemic Inflammation Induced by Extreme Weather Events and Air Pollution

Extreme weather events affect not only the inflammatory response in the local heart, but also other organs and tissues. It has been reported that inflammation in other organs can also affect cardiovascular health. Fine particles cross the epithelium and reach the deep pulmonary tissue. Macrophages resident in the lung will recognize and try to engulf the particles. Besides, PM2.5 can mediate M1 polarization through the PP2A-mTOR-p70S6K/4E-BP1 pathway, resulting in severe inflammation [86]. Simultaneously, immune cells can secrete abundant inflammatory mediators such as tumor necrosis factor-alpha (TNF-α), interleukins (IL-1, IL-6, IL-8, etc.), interferons (IFN-γ), and chemokines to recruit more immune cells from the circulating blood. Excessive inflammatory mediators can not only injure pulmonary tissue but also lead to subclinical systemic inflammation, which can induce heart injury.

It has been widely reported that reducing macrophage accumulation can lead to improved outcomes in myocardial infarctions. In a mouse model of myocardial infarction, pulmonary inflammation was found to significantly increase pro-inflammatory cytokines in the blood circulation after exposure to air pollution particulate matter. Furthermore, targeted depletion of alveolar macrophages has been shown to significantly decrease macrophage accumulation in the infarcted area [87].

### 4.5 Hemostatic Imbalance Caused by Extreme Weather Events and Air Pollution

Hemostasis and anticoagulation systems are essential for maintaining the integrity of the vasculature and the blood flow. Hemostasis promotes thrombus formation to prevent bleeding and repair injured vessels. However, excessive or inappropriate thrombosis can reduce blood supply and lead to ischemia in the affected tissues. In contrast, the anticoagulation system inhibits excessive thrombosis and promotes thrombolysis. Thrombosis and thrombolysis are complex physiological mechanisms involving numerous factors such as endothelial cell dysfunction, platelets, von Willebrand factor (vWF), coagulation factors, tissue factor, plasminogen, tissue-type plasminogen activator (t-PA), and plasminogen activator inhibitor-1 (PAI-1).

Recent research suggests that extreme weather events can disrupt the delicate equilibrium between hemostasis and anticoagulation, thereby increasing the risk of ischemic or thrombosis formation. During the Beijing Olympics, markers of thrombosis decreased among healthy young people due to the reduction in air pollution [88]. First, air pollutants injure the endothelial cells, resulting in the initiation of coagulation processes [89, 90]. Second, most studies have consistently indicated that short-term extreme weather events predominantly induce platelet activation, while long-term extreme weather events are associated with an increase in platelet count. Several human studies have confirmed that exposure to fine particle matter [91] and diesel exhaust [92, 93] is associated with the promotion of platelet activation and aggregation. In another study, mice were exposed to concentrated particulate matter for 2 weeks, and the results showed that the platelet counts increased by nearly 30% compared to those of the control groups. Besides, the binding of platelets to fibrinogen increased by 54%, indicating platelet activation [94]. Beyond platelets, air pollutants also target the coagulation cascade. To investigate the effects of PM on coagulation factors, plasma samples from healthy humans were collected to prepare platelet-poor plasma. The results revealed that traffic-related ultrafine particles could induce the formation of the thrombin by activating protease factors XII (FXII) and XI (FXI) [95]. In response to this pro-coagulant state, the fibrinolytic system is activated to dissolve thrombi and restore blood flow. At the same time, PM2.5 can induce phosphatidylserine externalization on red blood cells, thereby enhancing procoagulant activity and increasing the risk of thrombosis [96].

Air pollutants also dysregulate the fibrinolytic system, thereby potentially increasing the risk of thrombosis and organ ischemia. As an essential component of fibrinolysis, the t-PA level decreased among patients with stable coronary artery disease after exposure to diesel exhaust air. Furthermore, these patients exhibited a higher heart rate and severe ST-segment depression after exercise, indicating severe myocardial ischemia [97]. These mechanisms account to some extent for the increase in ischemic heart disease after exposure to high air pollution.

Although there are differences in the outcomes, ambient temperature can also impact thrombosis formation. Large population cohort studies have found that venous thrombosis formation is related to high ambient temperature [98]. Thermoneutral immersion in warm water bathing can decrease the activity of plasminogen activator inhibitor-1 and increase platelet count after adjusting for plasma volume changes caused by hemoconcentration [99]. However, the clinical data revealed an unexpected increase in the mortality rate and hospitalization of ischemic disease during the cold season, while it exhibited a decrease during the warm season [100, 101]. Controlled human studies also reported that the risk of coagulation is higher after exposure to cold temperatures [102]. This difference may be attributed to the highly complex nature of temperature’s effect on thrombus formation, which involves multiple competing physiological pathways [103]. The dilation of blood vessels and increased blood flow can inhibit thrombosis formation, whereas vasoconstriction and blood stasis may promote it.

Research has also explored the effects of gaseous components on thrombosis. *In vitro* studies have found that gaseous pollutants, such as NO, CO, and H_2_S, function as endogenous gas mediators that participate in the regulation of hemostasis, platelet activation, and blood vessel relaxation [104]. However, population-based observational studies have yielded contradictory conclusions. Some surveys have reported an increased risk of thrombosis and cardiovascular disease associated with gaseous air pollutants [105], while others have found no such association [106, 107]. Unfortunately, there is a lack of controlled exposure evidence to comprehensively explore the link between environmental gaseous air pollutants and thrombosis.

### 4.6 Behavioral Changes Associated With Extreme Weather Events and Air Pollution

Extreme weather events, especially natural disasters, induce behavioral changes, such as smoking, drinking, drug abuse, medicine adherence, and reduced physical activity [108]. These unhealthy lifestyle habits significantly increase the risk of CVD. As mentioned before, survivors of Hurricane Katrina exhibited long-term increases in smoking, drug abuse, and medication non-adherence [109, 110]. Similarly, the use of cigarettes, alcohol, and marijuana increased in Manhattan, New York after the September 11th terrorist attacks [111, 112]. Besides, more obesity and higher hyper- low density lipoprotein (LDL) cholesterolemia were found after the Great East Japan Earthquake [113, 114]. It is well-known that unhealthy habits can have detrimental effects on cardiovascular health.

In the short term after a disaster, accessing medicine often becomes difficult. A report revealed that after Hurricane Katrina, 7% of hypertension patients did not bring their medication during evacuation, and 16% of patients reported a lack of access to medication refills [115]. Among patients with human immunodeficiency virus (HIV), research has also found that a lack of food security may lead to medication non-adherence. People also need to face food and shelter security issues after disasters. Furthermore, individuals may seek psychological comfort from smoking, drinking, and overeating due to the immense pressure after a disaster [116]. Cross-sectional studies have revealed that people who evacuated after the Great East Japan Earthquake are associated with unhealthy dietary habits [117, 118]. Unfortunately, these behaviors easily become addictive, and many people never quit.

Physical activity levels frequently decline after a disaster. At the same time, extreme weather events, such as heat waves and air pollution, discourage outdoor exercise. Current evidence suggests that exercising outdoors in areas with high levels of air pollution increases the risk of CVD [119]. Consequently, expert guidelines also recommend reducing outdoor exercise during periods of heavy air pollution [120].

### 4.7 Impaired Cardiac Development Due to Extreme Weather Events and Air Pollution

The heart is the first functional organ to form during the development of the embryo, and an abnormal heart development may result in pregnancy loss [121, 122]. Epidemiological surveys have indicated that extreme weather events can affect heart development and lead to congenital heart disease. The rate of congenital heart disease is significantly higher in high-altitude locations than in areas at lower altitudes [123, 124].

Moreover, research has found that air pollution significantly affects heart development. Exposure to air pollutants such as PM2.5, PM10, NO_2_, CO, and O_3_ during pregnancy is related to increasing congestive heart abnormalities, such as ventricular septal defects, and atrial septal defect, and patent ductus arteriosus [125, 126, 127, 128]. These effects are particularly pronounced during the first three months of pregnancy, which represents a critical period for cardiac development [129, 130].

Heatwave exposure has also been established as a risk factor. Another interesting study conducted in Uppsala, a Swedish study, linked 12,161 birth temperature records during gestation to adult death records. During the test period, 95% of the weekly temperatures fell below 17 °C, and the mean ambient temperature during gestation was 5 °C. The researchers analyzed how long the pregnancies had been exposed to warm weather (>13.5 °C, the warmest quintile of local temperatures), and the results showed that exposure to hot weather during gestation for every 6% increase in warming days increased the risk of ischemic heart disease in adulthood by 16% when individuals experienced cold spells [131]. This may indicate that ambient temperatures during gestation can affect the offspring’s ability to endure cold temperatures. Moreover, heatwaves during pregnancy can exacerbate the adverse effects of PM2.5 on the development of congenital heart disease [132, 133].

Besides, exposure to housing renovation [134], and living near main roads [135] are related to congenital heart disease. Furthermore, heavy metals such as titanium and lead can cross the placental barrier, thereby increasing the risk of congenital heart disease [136, 137].

## 5. Air Quality Improvements and CVD Outcomes

Extreme weather events and air pollution are interrelated challenges, posing a significant impact on global public health. During rapid industrialization, China faced serious environmental pollution, with the annual PM2.5 concentration reaching 54 µg/m^3^, far above the WHO recommended level of 10 µg/m^3^. Air pollution became the fourth major preventable disease risk factor contributing to the burden of disease in 2010, leading to about 25.2 million Disability Adjusted Life Years (DALYs) [138]. With increasing environmental pollution and related health problems, the Chinese government has taken a series of actions to improve the environment, providing strong evidence that large-scale environmental intervention policy can effectively reduce pollution and significantly lower mortality and morbidity of CVDs.

During the 2008 Olympic and Paralympic Games, Beijing, China implemented a series of measures to limit operations at industrial and commercial combustion facilities, which significantly reduced air pollutants. Rich *et al*. [88] revealed that the improvement in air quality altered biomarkers of inflammation and thrombosis even in healthy adults. Similarly, during the COVID-19 pandemic, China adopted strict lockdown measures to prevent the spread of the virus, which also reduced the factory activities and vehicle use, these measures also significantly improved the air quality [139, 140]. These changes also significantly reduced the morbidity and mortality of CVDs [141, 142, 143]. Huang *et al*. [138] reported that if air pollution is reduced to the levels observed during the Beijing Olympic by 2030, it could prevent about 439,000 coronary heart disease (CHD) deaths and gain 3,379,000 life years in urban China, which has health benefits of the same order of magnitude as the combined benefits of a 25% improvement in hypertension control and a 30% smoking reduction. Research results conducted in other countries also support these results [144].

These data highlight the risk of CVDs from environmental pollution, with the disease burden being no less than that from traditional factors such as hypertension and smoking. Therefore, it is crucial to prioritize reducing air pollution as a key measure to prevent CVDs. The practice from China provides valuable experience for the world and substantiates that human-driven mitigation policies can effectively reduce climate-related cardiovascular burdens.

## 6. Limitations and Future Direction

Epidemiological studies have provided valuable insights about the relationship between extreme weather events, air pollution, and cardiovascular diseases. However, research on the underlying pathophysiological mechanisms remains limited. Experimental studies at the molecular and cellular levels are urgently needed to elucidate the biological processes involved. Since most existing studies are observational, primarily time-series or ecological analyses, there is a risk of residual confounding from unmeasured individual-level factors such as baseline health status, indoor climate control, and physical activity, which are often not accounted for.

Future research should focus on generating higher-quality evidence through randomized controlled trials and long-term cohort studies to more precisely assess risk. And wearable portable air-quality sensors may improve the accuracy of individual-level environmental exposure assessment in patients [145]. In addition, inconsistencies in outcome definitions and significant variation in study quality hinder cross-study comparability and complicated meta-analyses. From the perspective of climate justice, it is also important to note that most current studies have been conducted in middle-income countries, which may limit the generalizability of findings to low-income populations. Therefore, greater support from international organizations is needed to fund research in low- and lower-middle-income countries, where the burden of climate-sensitive cardiovascular risk may be even greater.

## 7. Conclusion

In summary, extreme weather events exert multifaceted and often synergistic effects on cardiovascular health through both pathways directly reaching the heart and pathways affecting non-cardiac organs. The pathways directly reaching the heart involve the translocation of particle pollutants and microbial pathogens into cardiac tissues, which leads to direct injury. Among the pathways affecting non-cardiac organs, extreme weather events trigger a cascade of effects, including metabolic stress, autonomic dysregulation, oxidative damage, inflammation, thrombosis, behavioral changes, and developmental programming, ultimately leading to an elevated risk of cardiovascular disease. Older adults, individuals with pre-existing conditions, and socioeconomically vulnerable populations are particularly vulnerable to these impacts.

Furthermore, extreme weather events can interact with one another, compounding their health effects [146]. For instance, heatwaves can promote the formation of O_3_ while dust storms increase the concentration of particle matter. Besides, the local composition of particle matters also modulates the toxicity and health risks associated with dust storms. Xu *et al*. [147] demonstrated that heat waves and PM2.5 act synergistically to increase the mortality risk from myocardial infarctions. These findings highlight the necessity of integrated and multifaceted strategies to mitigate the compounded hazards posed by extreme weather events.

Extensive evidence indicates that mitigating the effects of extreme weather events can significantly reduce the risk of CVDs. Looking forward, comprehensive policies, public health preparedness, and individual-level behavioral interventions are urgently needed to mitigate CVD risk. China’s success in reducing cardiovascular burdens through targeted air quality improvements and urban planning serves as a model for global implementation. Future research should focus on quantifying the effects of adaptive strategies and further elucidating mechanistic links to inform targeted, evidence-based prevention and resilience programs.
